# Ulnar dimelia variant: a case report

**DOI:** 10.1007/s10195-011-0146-y

**Published:** 2011-07-19

**Authors:** Javed Jameel, Abdul Qayyum Khan, Sohail Ahmad, Mazhar Abbas

**Affiliations:** Department of Orthopaedic Surgery, J.N. Medical College, A. M. U, Aligarh, 202002 Uttar Pradesh India

**Keywords:** Ulnar dimelia, Mirror hand, Pollicisation

## Abstract

We report a case of ulnar dimelia, commonly called mirror hand, in a 2-month-old female child who had restriction of elbow flexion and forearm rotation. There was no facial or other internal organ malformation. Radiographs revealed seven triphalangeal digits with double ulnae (one following the other) and absent radius. To the best of the authors’ knowledge, this is the first report of this mirror hand deformity in which fingers are symmetrical while duplicated ulnae are not.

## Introduction

Mirror hand (ulnar dimelia) is an extremely rare congenital anomaly of the upper limb [1]. Characteristically there is duplication of the ulna, absent radius and multiple fingers which are symmetrical around the midline. Over the years, there have been several case reports in the literature, and some variations of the deformity have been described.

We describe another variant of ulnar dimelia with multiple fingers symmetrical around midline along with duplication of the ulna, which was asymmetrical around midline, i.e. instead of facing each other, they were following each other.

## Case report

**Fig. 1:**
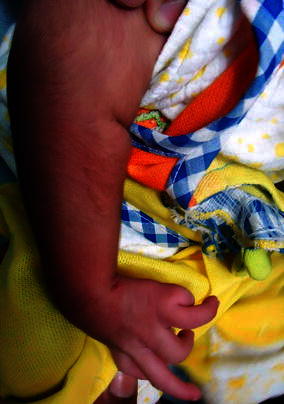
Flexion at elbow and radial deviation of wrist

**Fig. 2:**
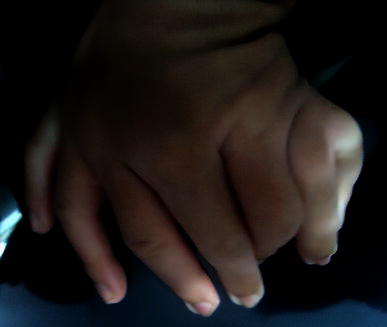
Dorsal aspect of hand with seven well-formed digits arranged symmetrically as mirror images on either side of a presumptive sagittal axis

**Fig. 3:**
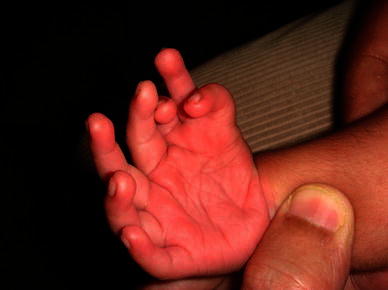
Palmar aspect of hand with seven well-formed digits arranged symmetrically as mirror images on either side of a presumptive sagittal axis

**Fig. 4:**
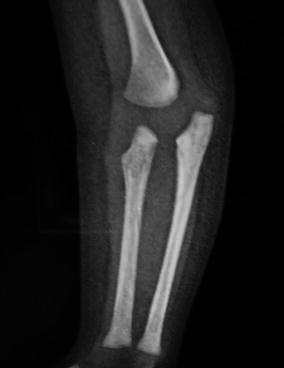
Radiograph of affected limb showing two ulnae following each other with broadened distal end and absent radius

**Fig. 5:**
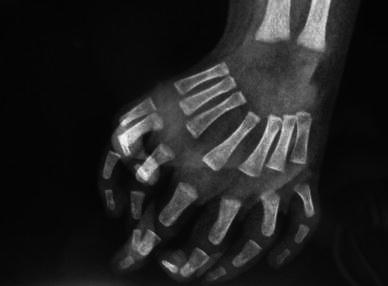
Radiograph of hand shows seven triphalangeal digits with seven corresponding metacarpals all in one plane

A 2-month-old female child was brought to Orthopaedic OPD with stiffness of elbow and congenital deformities of right hand. She was second child, born out of a non-consanguineous marriage by full-term normal vaginal delivery. There was no family history of congenital disorders, and antenatal period was uneventful. Examination revealed shortening of right upper limb with slight flexion at elbow and radial deviation of wrist (Fig. 1). The hand had seven well-formed digits arranged symmetrically as mirror images on either side of a presumptive sagittal axis (Figs. 2, 3). Passive movements at shoulder were normal; elbow had fixed flexion deformity of 20° with further flexion of 30°. Supination and pronation was grossly restricted at forearm; wrist movement was from 15° to 45° of palmar flexion. Child was able to flex and extend the fingers and occasionally grasped object between the fingers (although exact finger function could not be assessed as the child was too small). Function of opposite limb was essentially normal. Radiographs of affected limb show two ulnae, one following the other, with broadened distal end and absent radius (Fig. 4). The hand bears seven triphalangeal digits with seven corresponding metacarpals all in one plane (Fig. 5). We planned gentle manipulation at wrist and elbow, followed by serial corrective cast application at weekly intervals to correct as much deformity of wrist and elbow as possible. Correction of deformity can be maintained by suitable orthosis until the child reaches 1 year of age. After 1 year of age, staged surgeries can be planned because by that time the limb size becomes reasonably large for corrective and reconstructive surgeries and also the child can be trained well in use of hand and muscle training of pollicised finger. Our aim was to obtain useful elbow flexion, forearm rotation and reconstruction of thumb from the functional point of view. In the first stage, soft tissue distraction would be achieved by application of radial distractor across the wrist to correct wrist flexion and radial deviation. Second-stage surgery would be planned after correction of wrist deformity to assess full excursion of long flexors of fingers. First, we would achieve maximum flexion of elbow by excising about 1 inch of bone from proximal part of the radially placed extra ulna, followed by reconstruction of the collateral ligament and soft tissues to the proximal part of the normally placed ulna. Then we would excise two radial-most fingers with preservation of the skin flap from the middle finger with release of the first web. Shortening osteotomy at the level of the metacarpal neck of the remaining radial digit would be done, and the metacarpal head rotated for about 80° of apposition to make it thumb and fixed by two K-wires. A cortical bone graft from the amputated metacarpal bone would be pressed between the second metacarpal bone and that of the pollicised digit to fix it in abduction, and the skin flap would be transposed to first web. Post-operatively the limb would be kept in long arm splint with elbow flexed, wrist extended and thumb abducted for 4–6 weeks. Gradual passive physiotherapy would be started after removal of K-wires and splint.

## Discussion

Mirror hand (ulnar dimelia) is an extremely rare congenital anomaly of the upper limb [1]. Characteristically there is duplication of the ulna with both ulnae facing each other [2, 3], absent radius and multiple fingers which are symmetrical around the midline. There are few descriptions of mirror hand deformity with different variants in the literature. Al-Qattan and Al-Thunayan [4] proposed a classification of mirror hand deformity based on presence or absence of other congenital anomalies and the type of forearm bones present. Type 1 includes ulnar dimelia, type 2 includes two ulnae and one radius, type 3 includes one radius and one ulna, type 4 includes associated anomalies (syndromic) and type 5 includes multiple hands on a single limb. In the literature several variants of mirror hand deformity have been described. Multiple fingers with one ulna and one distinct radius [5], multiple fingers with two ulnae (one vestigial) and one radius [6] and multiple fingers with two ulnae (one vestigial) and one radius with duplicated proximal humerus [7] have been described. In the literature, no case has been reported so far with type 1 mirror hand variant, in which complete duplication of hand with seven fingers and double ulna occur and one ulna follows the other, instead of facing each other as mirror image.

Management of this condition is directed at achieving a functional and aesthetic upper limb and involves multiple, complex operations. Jafari performed pollicisation after excision of three digits in a case of ulnar dimelia with eight digits in a 20-year-old patient [7]. Avadis, in a 7-year-old patient of ulnar dimelia with seven digits, performed single-stage surgery involving pre-axial ulnar excision, excision of two radial digits, pollicisation and release of wrist flexors and achieved acceptable functional and cosmetic result [8]. Afshar also performed pollicisation after excision of one digit in a six-digit child of 4 years of age with ulnar dimelia [9].

In this patient, because of unusual duplication of ulna, the radial ulna seems to be non-articulating with trochlea, hence excision of proximal end of radial ulna along with repair of collateral ligament and soft tissues to the proximal part of the normally placed ulna by antero-lateral approach would give a reasonable result regarding elbow flexion and forearm rotation.

## Ethical considerations

Informed consent was taken from the parents of the patient prior to inclusion in the study. The study was performed according to the Declaration of Helsinki, and the Institutional Ethical Board approved it.
